# Are healthcare workers more likely than the general population to consult in primary care for an influenza‐like illness? Results from a case‐control study

**DOI:** 10.1111/irv.12750

**Published:** 2020-05-06

**Authors:** Arnaud Peytremann, Nicolas Senn, Yolanda Mueller

**Affiliations:** ^1^ Center for Primary Care and Public Health (Unisanté) University of Lausanne Lausanne Switzerland

**Keywords:** epidemiology, human, influenza, occupations, prevention and control, primary health care

## Abstract

**Background:**

Healthcare workers are at increased risk of contracting influenza. However, existing studies do not differentiate professional categories or domains of the healthcare system that are most at risk.

**Methods:**

This case‐control study compared proportions of patients with professional activity in the healthcare system between cases consulting their primary care physician for an influenza‐like illness (ILI) and controls from the general patient population of the same practices of the Swiss sentinel network. Influenza was confirmed by rRT‐PCR in a subset of practices. Analysis used a mixed logistic regression model, including age and sex as potential confounders.

**Results:**

During the 2018/2019 influenza surveillance season, out of 4287 ILI cases and 28 561 controls reported in 168 practices, 235 (5.5%), respectively 872 (3.1%), were active in the healthcare system. After adjustment, being active in health care increased the odds of consulting for an ILI (OR 1.66, 95% CI 1.40‐1.97). The association was strongest for physicians and nursing aides. In terms of work setting, odds of consulting for ILI were increased for professionals of almost all healthcare settings except home‐based care.

**Conclusion:**

Individuals active in the healthcare system were more likely to consult their primary care physician for an influenza‐like illness than for another reason, compared with individuals not active in the healthcare system. These results warrant further efforts to understand influenza transmission in the healthcare system at large.

## 1 INTRODUCTION

Healthcare workers are at increased risk of influenza infection compared to non‐HCW.^1^, ^2^, ^3^ For example, influenza‐like illness (ILI) among Italian medical residents peaks earlier compared to the general population.^3^ General practitioners (GPs) in particular have been shown to have high levels of basic immunity to influenza, probably resulting from frequent contacts with influenza viruses in the past.^4^

Already during the 1918 influenza pandemic, social class based on occupation had an impact on mortality.^5^ Occupation of influenza cases has been explored in more details during the 2009 H1N1 pandemic. In a study conducted in four American states, the proportion of healthcare workers was three times higher among laboratory‐confirmed influenza cases compared to its proportion in the general workforce.^6^ In a Spanish matched case‐control study, being a healthcare worker was associated with consulting as an outpatient for influenza.^7^

However, existing studies of influenza risk based on occupation do not differentiate between the different settings of the healthcare system, such as hospitals, residential homes, physician practices. Direct transmission from healthcare workers has been documented,^8^ but whether patients acquire influenza mostly from other patients or from healthcare workers is still debated.^9^, ^10^

Most of the work on healthcare‐associated influenza has been conducted in hospitals^11^ or long‐term care institutions. In hospitals, a significant proportion of influenza infections is acquired during admission.^12^ Patients visiting the emergency department for another reason than influenza during the influenza season have an increased risk of contracting influenza compared with community controls.^13^ In an outpatient setting, one retrospective cohort study among children aged two to five years old reported an increased risk of 36% (incidence rate ratio 1.36; 95% CI 1.22‐1.52) of presenting for an ILI visit in the 8 days after a non‐ILI visit to a pediatric clinic.^14^

Our research question was whether being professionally active in the healthcare system (exposure) increases the risk of influenza infection, assessed by consulting a primary care practitioner for influenza‐like illness (outcome). We assumed that healthcare workers would mostly consult their primary care practitioner in case of influenza‐like illness. Therefore, we estimated the association between seeking consultation for an influenza‐like illness or having confirmed influenza, and being professionally active in the healthcare system, differentiating by type of profession and work setting.

## 2 MATERIALS AND METHODS

This unmatched case‐control study was conducted within the Swiss national sentinel surveillance system (Sentinella) during the 2018‐2019 influenza surveillance season. Sentinella is a network of approximately 165 primary care physicians (general internal medicine specialist or pediatricians), maintained by the Swiss Federal Office of Public Health (SFOPH) since 1986 for the purpose of influenza surveillance. During the influenza surveillance season (epidemiological week 40 to 16), participating physicians declare on a weekly basis every case of influenza‐like illness, defined as a history of fever (>38°C), generally of abrupt onset, and presence of either sore throat or cough. Nasopharyngeal swabs are performed in a subset of practices, allowing identification of circulating strains in Switzerland by the National Reference Center of Influenza. Confirmed influenza cases are defined as ILI cases with positive nasopharyngeal swabs by rRT‐PCR. In order to obtain a denominator for ILI incidence, physicians report the daily number of patient contacts and, twice a year for a duration of two weeks, detailed patient‐contact information with documentation of age and sex. ILI incidence by number of inhabitants is extrapolated by triangulating the proportion of ILI per number of patient contacts with the number of consultation per individual, obtained from national statistics such as the Swiss Health Survey.

We used two different sets of cases in our study. First, cases were defined as all ILI cases, reported to Sentinella during the influenza surveillance period (October 2018 [week 40] to April 2019 [week 16]). In a second analysis, we restricted cases to confirmed influenza cases by rRT‐PCR. As controls, we used the patient contacts reported by physicians during week 11 and 12, 2019, minus ILI cases (patients with same sex and year of birth declared both as case and control within same week in same practice). Both for cases and controls, we added to the existing data collection a question about professional activity in the healthcare system, understood as the part of the health system providing health care to patients. Professional activity corresponded to the International Labour Office definition of occupied labor force. If professionally active, we further enquired about type of profession and work setting. Type of profession was categorized based on the International standard classification of occupations (ISCO version 08), simplified in eight categories relevant for the healthcare system, and based on the type of contact with patients: (1) physicians; (2) nurses; (3) nursing aides/personal care workers; (4) medical assistant or paramedics; (5) physical, occupational, or psycho‐therapist; (6) laboratory or radiology technician, pharmacy assistant; (7) pharmacist or dentist; (8) administrative personal; (9) other; and (10) unknown. Work setting was categorized as: (1) private practices; (2) hospital; (3) pharmacy; (4) at‐home care; (5) nursing home; (6) reeducation center; (7) dentist or therapist practices; (8) radiology or laboratory center; (9) office space; (10) other; and (11) unknown. In case of missing information about professional activity, data of people born before 1954 and after 2003 were recoded as “not active,” and the remaining “missing” recoded as unknown.

For both cases and controls, the following variables were obtained from the routinely collected Sentinella: week, age, sex. In addition, for ILI cases we collected whether the swab was sent to the reference laboratory, and rRT‐PCR result. At practice level, we obtained the region and total number patient‐physician contacts during influenza surveillance season. The project made full use of the quality assurance system of Sentinella. Declaring GPs received instructions about data collection, with main messages reinforced by regular Newsletters. Predefined checks in electronic data entry diminished the risk of data entry errors. The Sentinella program Commission, consisting of regional representatives of declaring physicians, Swiss family medicine institutes, and the SFOPH, reviewed the study protocol and data collection forms.

Analysis of this case‐control study was based on a mixed logistic regression model, taking into account the clustering by practice by including a random intercept. We considered age and sex as potential confounders, because age was associated with both types of profession and ILI incidence, and sex was associated with types of profession, as well as possibly associated with ILI incidence and health‐seeking behavior in case of ILI. Profession and work setting of patients active in the healthcare system were compared to those not active, excluding those with unknown or missing activity information (complete case analysis). If active, other professions with <5% of total and unknown profession were regrouped into a single category. If active, but profession, respectively, work setting, was missing, it was recoded as unknown. For confirmed influenza cases, the dataset was restricted to practices where swabs were performed. Separate models were used for activity in the healthcare system in general, categories of professional activity if active in the healthcare system, and categories of work settings, because of collinearity between these variables. In a sensitivity analysis, we repeated the model for activity in the healthcare system, setting all missing data to “inactive,” To examine possible over‐ or underrepresentation of some professions among controls, we compared the proportion of individuals active in each professional category among subjects aged 15‐64 years old with national occupational statistics.^15^ We used the Stata 15 software for all analyses.

The investigators had access only to anonymized data. Neither additional health‐related data nor biological material was collected specifically for the study. As such, the project was not under the scope of the Swiss human research law (LRH) and did not require formal ethical review.

## 3 RESULTS

During the 2018/2019 influenza surveillance season, there were 4287 ILI cases reported from 168 practices, out of which 346 were confirmed for influenza from the 79 practices swabbing ILI cases. During weeks 11 and 12, 28 561 controls were recorded, reduced to 15 463 after restricting the dataset to practices doing swabs.

The median age for the ILI cases was 33 (12‐52, 95% CI), compared with 52 (27‐71, 95% CI) for controls (Table 1). There were slightly more females among controls than among ILI cases (52.7% vs 50.2%, *P* = .001). Of the total, ILI cases 235 (5.5%) were working in the healthcare system, compared to 872 (3.1%) for controls. Professional activity was unknown for 546 (12.7%) ILI cases and 2865 (10.0%) of controls.

**Table 1:** Sample characteristics of influenza‐like illness (ILI), respectively, rRT‐PCR‐confirmed influenza cases, and controls representing the general patient population of primary care practices of the Swiss sentinel network Sentinella, 2018‐2019 influenza surveillance season

	Cases (ILI)	Controls	Cases (confirmed influenza)	Controls
N observation	N = 4287	N = 28 561	N = 346	N = 15 463
Median age in years (IQR)	33 (12‐52)	52 (26‐71)	35 (15‐55)	54 (25‐72)
N female (%)	2147 (50.1)	15 047 (52.7)	173 (50.0)	8174 (52.9)
Active in the healthcare system*				
Yes	235 (5.5)	872 (3.1)	23 (6.7)	434 (2.8)
No	3506 (81.8)	24 824 (86.9)	298 (86.1)	13 478 (87.2)
Unknown	546 (12.7)	2865 (10.0)	25 (7.2)	1 551 (10.0)

Note

* Missing activity and born before 1954 and after 2003 recoded as “not active”; otherwise recoded as unknown.

Being active in the healthcare system was associated with increased odds of consulting for an ILI (crude OR 1.91, 95% CI 1.65‐2.21; Table 2). The associations persisted after adjustment for age, sex, and inclusion of a random intercept for practice (Adj OR 1.66, 95% CI 1.40‐1.97). The association was strongest for the physicians (Adj OR 2.85, 95% CI 1.47‐5.53) and nursing aides (Adj OR 2.01, 95% CI 1.42‐2.85). Odds were also increased for administrative staff and for other or unknown profession. After adjustment, we found no increased odds for nurses nor for medical assistant and paramedical staff.

**Table 2:** Association between being active in the healthcare system and consulting for an influenza‐like illness (ILI)

	Cases (ILI) N = 3741	Controls (ILI) N = 25 696	Crude OR (95% CI)	Adjusted OR (95% CI)
	n (%)	n (%)		
Not active in the healthcare system	3506 (93.7)	24 824 (96.6)	1	1
Active in the healthcare system	235 (6.3)	872 (3.4)	1.91 (1.65‐2.21)	1.66 (1.40‐1.97)
Profession if active in the healthcare system				
Nurse	61 (1.6)	259 (1.0)	1.67 (1.26‐2.21)	1.28 (0.95‐1.74)
Nursing aide	54 (1.4)	156 (0.6)	2.45 (1.79‐3.35)	2.01 (1.42‐2.85)
Medical assistants/ paramedics	24 (0.6)	66 (0.3)	2.57 (1.61‐4.11)	1.46 (0.88‐2.44)
Administrative staff	17 (0.5)	65 (0.3)	1.85 (1.08‐3.16)	1.84 (1.02‐3.30)
Physician	14 (0.4)	42 (0.2)	2.36 (1.29‐4.33)	2.85 (1.47‐5.53)
Occupational, physical therapy, dietitian	7 (0.2)	52 (0.2)	0.95 (0.43‐2.10)	0.96 (0.41‐2.24)
Laboratory and radiology technicians, pharmacy assistants	8 (0.2)	14 (0.1)	1.77 (1.32‐2.36)	1.95 (1.40‐2.72)
Pharmacist, dentist	2 (0.1)	14 (0.1)		
Other	31 (0.8)	101 (0.4)		
Unknown	17 (0.5)	74 (0.3)		
Work setting if active in the healthcare system				
Nursing home	76 (2.0)	198 (0.8)	2.72 (2.08‐3.55)	2.06 (1.53‐2.78)
Hospital	51 (1.4)	187 (0.7)	1.93 (1.41‐2.64)	1.66 (1.18‐2.32)
Private practice	31 (0.8)	80 (0.3)	2.74 (1.81‐4.16)	2.26 (1.43‐3.58)
Home‐based care	13 (0.4)	56 (0.2)	1.64 (0.90‐3.01)	1.53 (0.79‐2.94)
Administration	7 (0.2)	8 (0.0)	1.29 (0.99‐1.69)	1.24 (0.92‐1.67)
Pharmacy	5 (0.1)	18 (0.1)		
Dentist, physical, occupational therapy	5 (0.1)	31 (0.1)		
Radiology, laboratory	2 (0.1)	18 (0.1)		
Rehabilitation	1 (0.0)	19 (0.1)		
Other	15 (0.4)	90 (0.4)		
Unknown	29 (0.8)	167 (0.6)		

Note

Missing activity excluded. Model adjusted for age (linear and quadratic), sex and cluster effect by practice. Unknown or missing activity excluded.

In terms of work setting, we found increased odds of consulting for ILI for professionals of almost all healthcare settings except home‐based care. The association was strongest for those working in private practices (Adj OR 2.26, 95% CI 1.43‐3.58) and nursing homes (Adj OR 2.06, 1.53‐2.78). It was also increased, to a lesser degree, for professionals working in hospitals. It was not significantly increased for workers in home‐based care and other healthcare settings.

Results for PCR‐confirmed influenza, although based on a limited number of cases, were consistent with results obtained for ILI overall (Table 3). The odds of consulting for a confirmed influenza were particularly high among physicians (Adj OR 6.83, 95% CI 1.78‐36.1) and nursing aides (Adj OR 2.32, 95% CI 1.02‐5.29), and for staff active in private practices (Adj OR 4.53, 95% CI 1.65‐12.41), hospitals (Adj OR 2.56, 95% CI 1.05‐6.23), and nursing homes (Adj OR 2.44, 95% CI 1.08‐5.53). No significant associations were found between confirmed influenza and being an administrative staff or a staff active in another or unknown profession.

**Table 3:** Association between being active in the healthcare system and consulting for PCR‐confirmed influenza

	Cases (confirmed influenza) N = 321	Controls (confirmed influenza) N = 13 912	Crude OR (95% CI)	Adjusted OR (95% CI)
	n (%)	n (%)		
Not active in the healthcare system	298 (92.8)	13 478 (96.9)	1	1
Active in the healthcare system	23 (7.2)	434 (3.1)	2.40 (1.55‐3.70)	1.81 (1.13‐2.90)
Profession if active in the healthcare system				
Nurse	4 (1.2)	124 (0.9)	1.46 (0.54‐3.97)	1.15 (0.41‐3.23)
Nursing aide	7 (2.2)	100 (0.7)	3.17 (1.46‐6.87)	2.32 (1.02‐5.29)
Medical assistants/paramedics	2 (0.6)	37 (0.3)	2.44 (0.59‐10.19)	1.40 (0.32‐6.24)
Administrative staff	1 (0.3)	40 (0.3)	1.13 (10.15‐8.25)	1.24 (0.16‐9.55)
Physician	3 (0.9)	20 (0.1)	6.78 (2.00‐23.0)	6.83 (1.78‐36.1)
Occupational, physical therapy, dietician	0 (0.0)	29 (0.2)	NA	
Laboratory and radiology technicians, pharmacy assistants	0 (0.0)	22 (0.2)	3.23 (1.40‐7.45)	0.93 (0.93‐5.46)
Pharmacist, dentist	0 (0.0)	6 (0.0)		
Other	5 (1.6)	39 (0.3)		
Unknown	1 (0.3)	17 (0.1)		
Work setting if active in the healthcare system				
Nursing home	7 (2.2)	114 (0.8)	2.78 (1.28‐6.01)	2.44 (1.08‐5.53)
Hospital	6 (1.9)	88 (0.6)	3.08 (1.34‐7.11)	2.56 (1.05‐6.23)
Private practice	5 (1.6)	46 (0.3)	4.92 (1.94‐12.5)	4.53 (1.65‐12.41)
Home‐based care	0 (0.0)	32 (0.2)	NA	
Administration	1 (0.3)	7 (0.1)	1.47 (0.60‐3.60)	0.89 (0.35‐2.25)
Pharmacy	1 (0.3)	6 (0.0)		
Dentist, physical, occupational therapy	1 (0.3)	17 (0.1)		
Radiology, laboratory	0 (0.0)	7 (0.1)		
Rehabilitation	0 (0.0)	10 (0.1)		
Other	1 (0.3)	41 (0.3)		
Unknown	1 (0.3)	66 (0.5)		

Note

Missing activity excluded. Model adjusted for age (linear and quadratic), sex and cluster effect by practice. Unknown or missing activity excluded.

In sensitivity analyses, we considered all individuals with unknown or missing activity in the healthcare system as not active instead of excluding them from the logistic regression models (Table S1). All associations found in the main analysis were confirmed. Associations were also consistent when restricting the data to cases and controls to individuals aged 15‐64 years old (Table S2). Finally, to get a sense of the healthy worker bias present in our data, we compared the proportions of individuals working in different categories or work settings among our control population with available national statistics (Table S3). With the exception of nurses, all professional categories were rather underrepresented among controls. Comparing disease severity of ILI between healthcare workers (HCW) and non‐healthcare workers, there were 1.8% (4/218) clinical pneumonia among HCW, compared with 3.9% among non‐HCW (126/3149), a difference that was not significant even after adjustment for risk of complication and age in a logistic regression model (Adj OR for pneumonia among HCW 0.56, 95% CI 0.20‐1.54).

## 4 DISCUSSION

In this study, individuals active in the healthcare sector were more likely to consult their primary care physician for an influenza‐like illness, respectively, confirmed influenza, than for another reason. In terms of professional categories, the association was particularly strong for physicians and nursing aides. Surprisingly, being active either as an administrative staff or as any other or unknown profession in the healthcare system was also associated with an increased risk of consulting for an ILI. This could be due both to a higher risk of infection and to more sensitization in healthcare settings to abstain from work in case of ILI symptoms. In terms of work settings, private practices and nursing home particularly stood out, followed by hospitals.

The main limitation of this work is that health‐seeking behavior of health professional in case of ILI may differ from the general patient population. However, we have few reasons to believe that health professionals would consult more frequently for ILI, a rather mild illness in the active population, than for other health issues, which would have led to overestimation of the association. On the contrary, previous studies have shown that health professionals tend to minimize ILI symptoms and continue to work despite recommendations against this.^16^, ^17^, ^18^ There were not significantly less patients presenting with clinical pneumonia among healthcare staff. In addition, we recognize that it would have been preferable to sample controls from the patient population over the same time‐period as the cases, but this was not considered feasible within the sentinel set‐up, and would have probably resulted in many more missing data. By contrast with other professional categories, we found no association between being active as a nurse and consulting for ILI. However, nurses were also more represented among controls than other healthcare worker categories, which could have biased the result toward the null.

This is the first study to explore the question of healthcare setting‐associated influenza transmission from a primary care standpoint. Individuals active in the healthcare system appear to be overrepresented both among ILI and among confirmed influenza cases. The observed differences between professions and work settings could reflect different contact intensity between professionals and influenza‐infected patients, as well as differences in adhesion to infection prevention and control measures.

However, our results suggest that other professionals working in health care, for example administrative staff, may also be at increased risk of influenza. One could argue that individuals not in direct contact with patients do not pose a particular hazard for vulnerable patients. However, they may contribute to the overall burden of circulating viruses. Besides, these professionals may also in contact with patients, for example when working at reception desks. Decreasing circulation of influenza viruses in healthcare settings is likely to be beneficial to patients. Also, for their individual health, staff should be informed of their increased risk of influenza if this finding is confirmed.

Currently, apart from influenza vaccination, most specific influenza control measures such as mask wearing focus on droplet transmission. More attention to standard precautions, including hand hygiene, surface disinfection, and ventilation, may be required to prevent influenza in the healthcare workforce at large. Our results suggest that private practices and nursing homes could constitute weak spots of infection control. While efforts to increase staff vaccination coverage should be sustained, specific infection control recommendations targeting these settings should be developed, taking into account their specificities. To guide such recommendations, further studies on transmission modes and evidence on effective interventions should be directly generated in the relevant settings, and not extrapolated from hospitals. For example, a prospective cohort study among staff of primary care practices should be conducted to estimate infection rates without being confounded by differences in health‐seeking behavior.

While sentinel practices do not constitute a representative sample of all primary care practices, we have no reason to believe that Sentinella practices would be more or less likely to have health professionals among their patients than other private practices. Also, the Swiss sentinel network covers all six regions of the country, and the demographic structure of the adult patient population is overall similar to the one of Swiss practices.^19^ While these results cannot be used to extrapolate the proportions of professionals working in the healthcare system, we believe that the reported associations are valid. Still, we cannot exclude the possibility that health professionals were more likely to consult their physician for ILI, knowing that their physician was part of Sentinella. Overall, these findings certainly justify further attention to prevention of influenza transmission in the health system, particularly outside hospitals.

## Supporting information

Table S1‐S3
