# Supplementary material for: Are healthcare workers more likely than the general population to consult in primary care for an influenza‐like illness? Results from a case‐control study
Source: Influenza Other Respir Viruses. 2020 May 6;14(5):524–9. doi: 10.1111/irv.12750 (PMC7431651; doi:10.1111/irv.12750)
Supplement: Supplementary file 1 — Table S1‐S3 [file IRV-14-524-s001.docx]

**Supplementary material: sensitivity analyses conducted as part of the case-control study evaluating whether health care workers are more likely than the general population to consult in primary care for an influenza-like illness.**

Table S1. Association between consulting for an influenza-like illness (ILI), respectively a PCR-confirmed influenza, and professional activity in the health care system, **considering those with missing or unknown activitiy in the health care system as inactive**. Swiss Sentinella surveillance network, influenza surveillance season 2018-2019.

|  | ILI  (N=32,848) | | | Confirmed influenza  (N=15,805) | | | | |
| --- | --- | --- | --- | --- | --- | --- | --- | --- |
|  | AdjOR | (95% CI) | | AdjOR | | (95% CI) | | |
| Active in the health care system | 1.59 | | (1.35-1.88) | | 1.93 | | (1.21-3.06) | |
| Profession if active in the health care system |  | |  | |  | |  |  |
| - Nurse | 1.24 | | (0.92-1.68 | | 1.17 | | (0.42-3.27) | |
| - Nursing aid | 1.93 | | (1.34-2.71) | | 2.52 | | (1.12-5.67) | |
| - Medical assistant/paramedic | 1.39 | | (0.84-2.32) | | 1.49 | | (0.34-6.59) | |
| - Administrative staff | 1.61 | | (0.90-2.89) | | 1.24 | | (0.16-9.47) | |
| - Physician | 2.72 | | (1.41-5.25) | | 7.72 | | (2.06-28.9) | |
| - Occupational, physical therapy, dietician | 0.82 | | (0.40-2.13) | | NA | |  | |
| - Other/unknown profession | 1.91 | | (1.38-2.65) | | 2.44 | | (1.01-5.90) | |
| Work context if active in the health care system |  | |  | |  | |  | |
| - Nursing home | 2.07 | | (1.55-2.77) | | 2.65 | | (1.18-5.95) | |
| - Hospital | 1.64 | | (1.17-2.29) | | 2.80 | | (1.17-6.74) | |
| - Private practice | 2.10 | | (1.33-3.32) | | 4.57 | | (1.68-12.46) | |
| - Home-based care | 1.36 | | (0.71-2.61) | | NA | |  | |
| - Other/unknown contexts | 1.14 | | (0.85-1.54) | | 0.92 | | (0.36-2.33) | |

Table S2. Association between consulting for an influenza-like illness (ILI), respectively a PCR-confirmed influenza, and professional activity in the health care system, **dataset restricted to individuals aged 15 to 64 years old**. Swiss Sentinella surveillance network, influenza surveillance season 2018-2019.

|  | ILI  (N=13,573) | | | | Confirmed influenza  (N=5,885) | | |
| --- | --- | --- | --- | --- | --- | --- | --- |
|  | AdjOR | | (95%CI) | | AdjOR | (95%CI) | |
| Active in the health care system | 1.65 | (1.39-1.97) | | 1.76 | | (1.06-2.90) |  |
| Profession if active in the health care system |  |  | |  | |  |  |
| - Nurse | 1.31 | (0.96-1.78 | | 1.28 | | (0.45-3.65) |  |
| - Nursing aid | 1.91 | (1.34-2.73) | | 2.00 | | (0.82-4.86) |  |
| - Medical assistant/paramedic | 1.50 | (0.89-2.53) | | 1.20 | | (0.25-5.91) |  |
| - Administrative staff | 2.09 | (1.16-3.76) | | 1.34 | | (0.17-10.39) |  |
| - Physician | 3.08 | 1.54-6.15 | | 7.86 | | (1.89-32.63) |  |
| - Occupational, physical therapy, dietician | 0.79 | (0.31-2.04) | | NA | |  |  |
| - Other/unknown profession | 1.87 | (1.32-2.65) | | 2.19 | | (0.86-5.58) |  |
| Work context if active in the health care system |  |  | |  | |  |  |
| - Nursing home | 2.03 | (1.50-2.75) | | 2.40 | | (1.08-5.71) |  |
| - Hospital | 1.65 | (1.17-2.33) | | 2.40 | | (0.93-6.19) |  |
| - Private practice | 2.39 | (1.49-3.85) | | 5.20 | | (1.84-14.69) |  |
| - Home-based care | 1.51 | (0.77-2.94) | | NA | |  |  |
| - Other/unknown contexts | 1.20 | (0.87-1.64) | | 0.85 | | (0.32-2.26) |  |

Table S3. Comparison of **proportion of individuals aged 15 to 64 years old professionally active** in different professional categories in the health care system, between controls of a case-control study attending a primary care practice of the Swiss sentinel network and the general population.

|  | Controls aged 15 to 64 years old (N=14,088) | | n occupied active aged 15 to 64 ^a^ | % of the 15 to 64 resident population  (N=5,641,500) ^b^ |
| --- | --- | --- | --- | --- |
| Active in health care sector |  |  |  |  |
| - Yes | 817 | 5.80% | 379,478 | 6.73% |
| - No | 10,607 | 75.29% |  |  |
| - Unknown | 2,664 | 18.91% |  |  |
| Profession if active in health care sector |  |  |  |  |
| - Physician | 31 | 0.22% | 49,493 | 0.88% |
| - Nurse | 248 | 1.76% | 93,734 | 1.66% |
| - Nursing aid | 153 | 1.09% | 108,602 | 1.93% |
| - Medical assistant/paramedic | 62 | 0.44% | 44,626 | 0.79% |
| - Occupational, physical therapy, dietician | 47 | 0.33% | 35,392 | 0.63% |
| - Laboratory and radiology techinicians, pharmacy assistants | 40 | 0.28% | 31,086 | 0.55% |
| - Pharmacist, dentist | 14 | 0.10% | 16,545 | 0.29% |
| - Administrative staff | 61 | 0.43% |  |  |
| - Other | 96 | 0.68% |  |  |
| - Unknown | 65 | 0.46% |  |  |
| Work context if active in health care sector |  |  |  |  |
| - Private practice | 70 | 0.50% | 69,452 | 1.23% |
| - Hospital | 182 | 1.29% | 212,000 | 3.76% |
| - Pharmacy | 18 | 0.13% |  |  |
| - Home care | 52 | 0.37% | 50,744 | 0.90% |
| - Nursing home | 192 | 1.36% | 131,361 | 2.33% |
| - Rehabilitation | 19 | 0.13% |  |  |
| - Dentist, physio, ergo practices | 30 | 0.21% |  |  |
| - Radiology, laboratory | 18 | 0.13% |  |  |
| - Administration | 7 | 0.05% |  |  |
| - Other | 82 | 0.58% |  |  |
| - Unknown | 147 | 1.04% |  |  |

**^a^** Source of data: Swiss Federal Statistical Office. Profession: Structural survey 2017. § Work context: Structural data of medical practices and ambulatory centres 2017, hospital statistics 2017, statistics of medico-social institutions (SOMED) 2017, Home-care statistics 2017

^b^ Resident population by age: STATPOP.
